# Pathway-based joint effects analysis of rare genetic variants using Genetic Analysis Workshop 17 exon sequence data

**DOI:** 10.1186/1753-6561-5-S9-S45

**Published:** 2011-11-29

**Authors:** Pingzhao Hu, Wei Xu, Lu Cheng, Xiang Xing, Andrew D Paterson

**Affiliations:** 1 The Centre for Applied Genomics (TCAG) and Program in Genetics and Genome Biology, The Hospital for Sick Children, 101 College Street, Toronto, ON M5G 1L7, Canada; 2 Dalla Lana School of Public Health, University of Toronto, Health Sciences Building, 155 College Street, Toronto, ON M5T 3M7, Canada; 3 Department of Biostatistics, Princess Margaret Hospital, 610 University Avenue, Toronto, ON M5G 2M9, Canada; 4 Department of Computer Science and Statistics Department, University of Toronto, 40 St. George Street, Toronto, ON M5S 2E4, Canada

## Abstract

Pathway-based analysis has been recently used in joint tests of association between disease and a group of common genetic variants. Here we explore this idea for the joint effects analysis of rare genetic variants and their association with quantitative traits and disease. We accumulate multiple rare minor alleles in a genetic risk score for each individual in a given pathway; this score is then used to assess association with quantitative phenotypes and disease. We demonstrate that this approach may be better than studying single rare variants or a gene risk score for identifying individuals with significantly greater risk.

## Background

In the past few years, genome-wide association studies have been widely used to identify genetic risk factors for complex diseases. This analysis paradigm has made significant progress in many genetic studies. For example, so far, many variants have been discovered that are associated with common diseases, such as type 2 diabetes [1]. To date, however, the utility of genetic markers to improve disease risk prediction still explains only a small proportion of the genetic variance for many complex diseases. This missing heritability might be explained by common variants with weak effects and/or additional rare variants with strong or weak effects, acting additively and/or interacting with other genetic and environmental variants [2]. For the common variants explanation, multilocus-based genetic risk score and pathway-based methods have been developed. For example, the use of a multilocus genetic risk score has been proposed to evaluate the risk of breast cancer and its subtypes [3]. A pathway-based analysis strategy has been used to search for related genes and common single-nucleotide polymorphisms (SNPs) that contribute to Parkinson’s disease [4]. For rare variants, methods for statistical analysis are still limited. Here, we integrate the multilocus genetic risk score and pathway analysis strategies used for common variants to analyze rare genetic factors and evaluate their association with quantitative phenotypes and disease.

## Methods

### Data description

We use replicate 1 with 697 unrelated individuals from the Genetic Analysis Workshop 17 (GAW17) data. There are 24,487 autosomal SNPs. All SNPs and samples have a genotype call rate of 100%. We define rare SNPs as those with a minor allele frequency (MAF) less than 1%. For the phenotype data, there are three quantitative risk factors (Q1, Q2, and Q4), which are simulated as normally distributed phenotypes, and one disease risk factor, for which there are 209 case subjects and 488 control subjects. Genes influencing Q1 and the disease are primarily from the vascular endothelial growth factor (VEGF) pathway, whereas those influencing Q2 are primarily associated with cardiovascular disease risk and inflammation. There are no causal genes or pathways related to Q4 [5].

### Univariate SNP association analysis

For each rare SNP, we performed linear regression analysis for association between genotypes and Q1, Q2, and Q4 and logistic regression analysis for association between genotypes and disease status. We adjusted for sex, age, smoking, and population stratification by generating two dummy covariates for the three populations: Asian, African, and European. Genotypes were coded additively. We obtained the corresponding adjusted odds ratio (OR) and *P*-value for each rare SNP.

### Joint effects analysis of rare genetic variants

We analyzed the joint effects of rare genetic variants at both the gene and the pathway level. The objective was to test for association of an aggregation of rare minor alleles with quantitative phenotypes and disease by combing genetic information across multiple variants within a given gene or pathway, respectively. To do this, we first mapped rare SNPs to genes and pathways as follows.

In step 1, we mapped rare SNPs to genes. We obtained the nearest gene name for each rare SNP from the snp_info file provided by GAW17. In step 2, we mapped the genes from step 1 to the c2 curated canonical pathways (version 3) from the Broad Institute (http://www.broadinstitute.org/gsea/msigdb/). This database includes 888 gene sets collected from 186 pathways from the Kyoto Encyclopedia of Genes and Genomes (KEGG) (http://www.genome.jp/kegg/), 430 pathways from Reactome, 217 pathways from BioCarta among others. We kept only the pathways with at least five genes in our data set, which left 472 pathways for follow-up analysis. We note that each of these three databases includes the VEGF causal pathway. In the next step, we defined a genetic risk score for each individual as the count of minor alleles of all rare variants in a given gene or pathway [6]. Finally, we performed linear and logistic regression analyses to test for the association of the count of minor alleles with the traits (Q1, Q2, Q4, and disease) in each gene and pathway, respectively, by adjusting for sex, age, smoking, and population stratification, similar to the univariate SNP association analysis.

## Results

**Figure 1:**
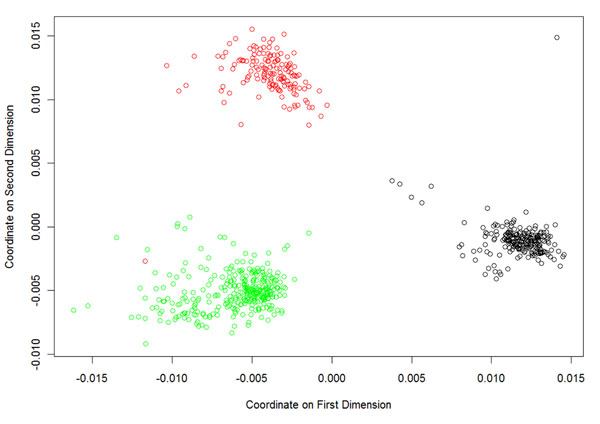
**Multidimensional scaling analysis of 697 samples from the 1000 Genomes Project**. We used 23,173 SNPs in the multidimensional scaling analysis and removed seven outlier samples (one European and six Africans) in the subsequent analysis. Red circles, Europeans; green circles, Asians; black circles, Africans.

We removed 1,314 SNPs that had a Hardy-Weinberg equilibrium test *P*-value smaller than 1 × 10^−6^ in control subjects, leaving 23,173 SNPs. We performed multi-dimensional scaling analysis using these remaining SNPs and identified seven outlier samples (Figure 1): One outlier is European (red circles) and clusters with the Asian group (green circles), and the other six outliers are African (black circles) and are separate from the major African cluster (lower right-hand corner). The seven outlier samples include five control and two case subjects. We removed these outliers in subsequent analyses.

We focused on 18,094 rare variants (MAF < 1%) from the 23,173 SNPs that passed quality control. Overall, we did not find any rare variants with significant association for Q2, Q4, and disease at the Bonferroni-corrected significance level of 0.05 (corresponding to an unadjusted *P*-value of 2.8 × 10^−6^). We did find three rare variants that were significantly associated with Q1 (Table 1). One of these (C13S524) is in causal gene *FLT1*, whereas the other two are not in any causal gene.

**Table 1:** Identified significant rare genetic variants for Q1

SNP	Chromosome	Position	MAF	*P*-value	*β* (standard error)	In causal gene?
C13S524	13	27,899,915	0.0043	2.33 × 10^−7^	1.92 (0.37)	Yes (*FLT1*)
C2S2355	2	112,864,155	0.0087	6.43 × 10^−7^	1.31 (0.26)	No (*RGPD8*)
C2S2174	2	107,855,174	0.0094	2.66 × 10^−6^	1.04 (0.22)	No (*RGPD4*)

The 18,094 rare variants mapped to 2,439 genes. Of these 2,439 genes, 911 had 1 rare SNP in each gene, 655 had 2–5 rare SNPs, and 873 had more than 5 rare SNPs. The joint effects analysis of rare genetic variants at the gene level did not identify significant association for Q2, Q4, or disease at the Bonferroni-corrected significance level of 0.05 (corresponding to an unadjusted *P*-value of 2.1 × 10^−5^). We found four genes that were significantly associated with Q1 (Table 2), two of which (*FLT1* and *KDR*) were causal genes and two of which (*RGPD8* and *EPHB1*) were not causal genes.

**Table 2:** Significant association of genes with Q1

Gene	Chromosome	Number of rare SNPs	*P*-value	*β* (standard error)	Causal genes?
*RGPD8*	2	3	2.55 × 10^−8^	1.30 (0.23)	No
*FLT1*	13	25	7.37 × 10^−8^	0.67 (0.12)	Yes
*KDR*	4	14	8.52 × 10^−7^	0.88 (0.18)	Yes
*EPHB1*	3	6	1.53 × 10^−5^	0.81 (0.19)	No

The 2,439 genes that include at least one rare variant were mapped to 472 canonical pathways. We did not find significant association of pathways with either Q2 or Q4 at a Bonferroni-corrected significance level of 0.05 (corresponding to an unadjusted *P*-value of 1.0 × 10^−4^). The most significant pathway for Q2 was Reactome Phase II Conjugation (*P* = 6.5 × 10^−4^), which did not include causal genes.

As described before, each of the three pathway databases (KEGG, Reactome, and BioCarta) includes the VEGF causal pathway for Q1 and disease. We report the association results of this causal pathway in Table 3, which shows that the VEGF causal pathway in the three databases is significantly associated with Q1 but is significantly associated with disease only in the Reactome and BioCarta databases. The likely reason for this is that the VEGF pathway genes in KEGG include fewer causal genes for either Q1 or disease than those in either Reactome and BioCarta. All 5 genes of the VEGF pathway in the Reactome database are causal genes for Q1; 9 of 11 genes of the VEGF pathway in the BioCarta database are causal for either Q1 (6) or disease (3); and only 7 of the 17 genes in the VEGF pathway in KEGG are causal for either Q1 (2) or disease (5).

**Table 3:** Association of rare SNPs in the VEGF pathway with traits in three databases

Trait	Database	Number of rare SNPs	Number of genes in GAW17 data set	Number of causal genes	*P*-value	Rank of significance
Q1	Reactome	53	5	5	1.77 × 10^−15^*	1
	BioCarta	95	11	6	6.81 × 10^−14^*	2
	KEGG	66	17	2	1.83 × 10^−7^*	11

Disease	Reactome	53	5	0	4.95 × 10^−5^*	3
	BioCarta	95	11	3	4.69×10^−5^*	2
	KEGG	66	17	5	2.17×10^−4^	4

* Significant at corrected significance level of 0.05.

**Figure 2:**
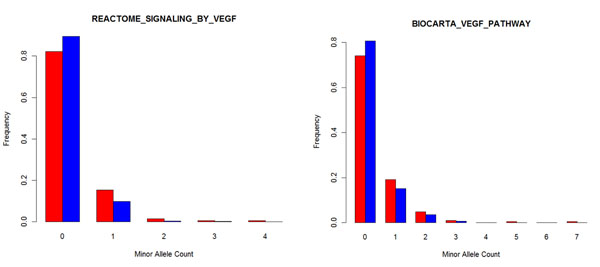
**Distribution of minor allele counts in case and control subjects**. The frequencies of minor alleles in different bins were estimated for the VEGF pathway in the Reactome and BioCarta databases. Red bars, case subjects; blue bars, control subjects.

Because the VEGF pathway is significantly associated with disease, as shown in Table 3, we present in Figure 2 the distributions of minor allele count in case and control subjects for this pathway in the BioCarta and Reactome databases. The figure clearly shows that the rare minor allele count is significantly greater in case subjects than in control subjects.

To evaluate whether the significance of the causal pathways in different databases is driven by only one rare SNP out of each gene in a given pathway, we did a leave-one-out cross-validation (LOOCV). To do this test, for each pathway we first removed one rare SNP from each gene and then recalculated the genetic risk score for each individual as the accumulation of minor alleles for all rare variants in a given pathway by excluding the rare SNP. Finally, we performed the regression analysis described earlier. We repeated these steps for all the rare SNPs in a given pathway. Table 4 shows the results for the VEGF causal pathways for Q1 and disease. The table clearly shows that the significance of the VEGF causal pathway for Q1 is not affected by one single rare SNP but by multiple rare minor alleles in the pathway, whereas the disease risk is driven by a small number of truly associated rare SNPs. For example, all five SNPs in the BioCarta database and all six SNPs in the Reactome database are in causal genes for either Q1 or disease.

**Table 4:** LOOCV rare SNP results for VEGF pathway with trait in three databases

Trait	Database	Number of rare SNPs	*P*-value (all SNPs)	One SNP removed at a time	
	
				Number of times *P* ≤ 1 × 10^−4 a^	Number of times *P* > 1 × 10^−4^
Q1	BioCarta	95	6.81 × 10^−14^	95	0
	Reactome	53	1.77 × 10^−15^	53	0
	KEGG	66	1.83 × 10^−7^	66	0

Disease	BioCarta	95	4.69 × 10^−5^	90	5
	Reactome	53	4.95 × 10^−5^	47	6
	KEGG	66	2.17 × 10^−4^	1	65

^a^ At corrected significance level 0.05.

Because our analysis has narrowed the associations to the VEGF pathway in the three databases, we further evaluated the associations between SNPs in the pathway with Q1 and disease using a smaller Bonferroni correction based on the number of SNPs in the pathway in each of the three databases (see *P*-value column in Table 5). Based on the significance levels, we counted the number of true positives (causal SNPs significantly associated with Q1 or disease) and estimated the power for Q1 and disease in the three databases. Our results show that the two-stage approach (identifying the causal pathway and then detecting SNP associations in the causal pathway) has slightly larger power than trying to detect SNP associations directly for Q1 (see Tables 2 and 5).

**Table 5:** Power of rare SNPs in the VEGF pathway

Trait	Database	Number of rare SNPs	Number of causal rare SNPs	*P*-value^a^	Power (%) (true positives)
Q1	BioCarta	95	33	9.34 × 10^−4^	9.1 (3)
	Reactome	53	25	5.26 × 10^−4^	16.0 (4)
	KEGG	66	11	7.58 × 10^−4^	18.2 (2)

Disease	BioCarta	95	5	9.34 × 10^−4^	0 (0)
	Reactome	53	0	5.26 × 10^−4^	0 (0)
	KEGG	66	7	7.58 × 10^−4^	0 (0)

^a^ Significant at corrected significance level 0.05.

## Discussion and conclusions

In this study, we evaluated the associations between rare genetic variants and quantitative traits or disease status at the SNP, gene, and pathway levels. Overall, we did not find significant associations at the SNP, gene, and pathway levels for Q2 and Q4, but we found that the VEGF causal pathway is significantly associated with both Q1 and disease status. We also found that one causal SNP and two causal genes are significantly associated with Q1. We further confirmed that these enriched pathway signals are not driven by a single rare SNP but by multiple rare variants in multiple genes in the pathways for Q1. We assumed in our analysis that all minor alleles influenced each trait with the same direction of effect, because the minor allele in the simulated data is associated with higher means of quantitative traits (Q1 and Q2) and liability (disease). However, this assumption may not hold in real data, resulting in decreased power to detect causal genes. We have also observed that, although most of the causal genes for Q1 are not the causal genes for disease, those causal genes for Q1 actually play a key role in disease (such as the five genes of the VEGF pathway in the Reactome database). Our results show that a simple but efficient pathway-based analysis of rare genetic variants can identify potential genetic risk factors that were missed in the SNP- and gene-level analysis.

Our study does have some limitations. One is that our analysis was focused on only one simulated data set. A better strategy would be to analyze all simulated data sets. Another limitation is that we assumed that there was a linear relationship between the traits (Q1, Q2, and Q4) and the covariates used in the study. We observed that some of the covariates had a nonlinear relationship with the traits. Therefore nonlinear regression models may be more suitable. We will explore these models in detail in the future.

## Competing interests

The authors declare that there are no competing interests.

## Authors’ contributions

PH designed the study, performed the data analysis and drafted the manuscript. WX and ADP participated in designing the study. ADP supervised the study. All authors participated in the statistical analysis and helped to draft the manuscript. All authors read and approved the final manuscript.
